# Navigating the path to equitable rheumatologic care for underserved children with quality improvement

**DOI:** 10.3389/fped.2024.1426588

**Published:** 2024-10-15

**Authors:** Sheetal S. Vora, Sarah C. Mabus, Talia L. Buitrago-Mogollon

**Affiliations:** ^1^ Division of Rheumatology, Department of Pediatrics, Atrium Health Levine Children’s Hospital, Charlotte, NC, United States; ^2^ Department of Pediatrics, Wake Forest School of Medicine, Winston Salem, NC, United States; ^3^ Department of Pediatrics, Center for Advancing Pediatric Excellence, Atrium Health Levine Children’s Hospital, Charlotte, NC, United States; ^4^ Quality Management, Atrium Health, Charlotte, NC, United States

**Keywords:** health equity, specialty care, quality improvement, referral accuracy, care continuum

## Abstract

**Objective:**

The aim of this quality improvement project is to identify children with rheumatologic conditions to prevent delayed or missed diagnosis in underserved pediatric populations. Our focus is on prompt and accurate identification and subsequent treatment of rheumatologic symptoms in pediatric patients referred from Atrium Health safety-net primary care clinics that deliver care to families without private insurance, including those lacking insurance entirely.

**Methods:**

We collaborated with providers at one safety-net clinic to improve the processes of identification and subspecialty referral, resulting in an increase in the number of identified pediatric patients and referrals for these patients with potential rheumatologic disease. We used the Model for Improvement framework with rapid Plan–Do–Study–Act cycles and evaluated improvement with run and statistical process control charts.

**Results:**

We achieved improvement, with zero referrals in the previous 5 years for the targeted population increasing to 15 patient referrals within 1 year of project initiation. Despite this increase in referrals, the rheumatology clinic was able to see all priority patients within 20 business days from referral.

**Conclusion:**

An awareness of concerning rheumatologic symptoms in safety-net primary care clinics, combined with the use of both visual and decision aids, allows care teams to efficiently recognize and accurately refer patients needing specialty care.

## 1 Introduction

Rheumatologic conditions are notoriously difficult to diagnose because of myriad symptoms affecting multiple organ systems, evolving over time before a definitive diagnosis is reached (1). Given their limited resources arising from financial problems and logistical barriers, health literacy burden, and demands from other types of family crises, patients from lower socioeconomic backgrounds have historically been subjected to the challenges and resulting consequences of delayed diagnosis and access to subspecialist care (2–5).

Founded in 2011, the Atrium Health Levine Children's Specialty Center Rheumatology (LCSC-RC) division serves both clinic outpatients and hospital inpatients. In 2020, Atrium Health started focusing attention on rectifying care inequities. LCSC-RC recognized this as an opportunity to use a quality improvement (QI) methodology to improve patient care. This project builds upon a prior QI project (6) that expedited referrals based on symptoms, with the current project further exploring and improving access for children with fewer resources available to them.

## 2 Methods

### 2.1 Aim and measures

The aim of this project was to identify underserved children with rheumatologic conditions to prevent delayed treatment or missed diagnosis. Our focus was on prompt and accurate identification and subsequent treatment of rheumatologic symptoms in pediatric patients referred from four Atrium Health safety-net primary care clinics (PCCs). The proxy measures that were taken are listed as follows:

• A 10% increase in referrals needing rheumatologist evaluation for pediatric patients from four Atrium Health safety-net clinics to LCSC-RC from September 2020 to September 2021.
• A total of 80% of all referred LCSC-RC patients scheduled to be seen within 30 business days from referral.

We used the Model for Improvement with rapid Plan–Do–Study–Act (PDSA) cycles as a framework. At project initiation, the core team, comprised of a pediatric rheumatology physician lead, resident lead, QI coach, and data analyst, secured partial grant funding. Given the project's aim to improve the quality of care locally, the Institutional Review Board approved the project as a Quality Improvement Project.

### 2.2 Problem description

**Figure 1:**
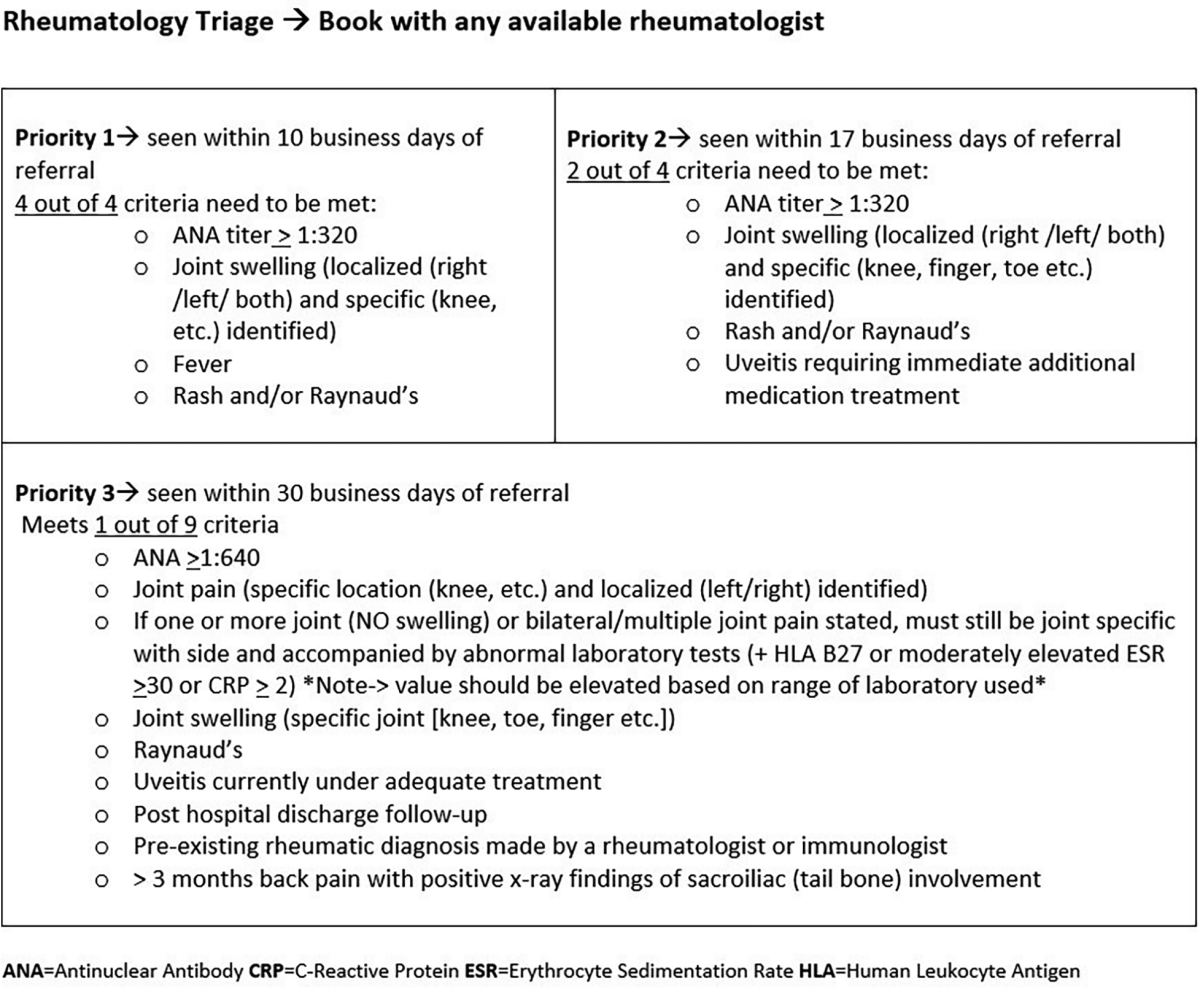
Triage Tool—Decision support for rheumatology referrals. Regardless of whether the patient is referred by a primary care provider or a specialist, the patient needs to have met the required number of criteria per priority designation.

To better understand the gap in pediatric rheumatologic care for those with limited resources, we reviewed data extracted from the electronic medical record (EMR) for pediatric patients who met all of the following criteria: (1) pediatric patients who received primary care services at Atrium Health between October 2015 and May 2020; (2) those living in either of two zip codes in the city designated as “public health priorities” due to higher rates of chronic diseases, infectious diseases, and deaths related to these conditions (7, 8); and (3) those presenting with symptoms indicative of rheumatologic disease. The third criterion was established previously to identify the group of symptoms most commonly found in referred patients, which ultimately led to a diagnosis of a rheumatologic condition, or at least required ongoing rheumatologic care (Figure 1, see priorities 1, 2, and 3) (6). The relevant symptom categories were joint pain, unexplained fever lasting >10 days, and positive antinuclear antibody (ANA). Four safety-net clinics emerged as seeing a large majority of patients from this search. These sites became our target population. Safety-net clinics deliver care to families without private insurance, including those lacking insurance entirely.

### 2.3 Baseline data

The review of our baseline data spanning 5 years revealed that of 2,239 qualifying patients, 1,929 (86%) were seen in four Atrium Health safety-net clinics and only five children (0.2%) ultimately consulted with a pediatric rheumatologist in our system. It was unknown whether the remainder of the qualifying patients were seen by a pediatric rheumatologist outside Atrium or whether the symptoms ultimately resolved without treatment.

As the COVID-19 pandemic erupted placing strict limitations on in-person interactions and severe burden on the healthcare system, the team pivoted from targeting all four safety-net clinics to only the main pediatric safety-net PCC where 858 of the 2,239 eligible patients were seen yet zero referred.

The PCC was also the best option due to its locational advantages: it was juxtaposed one block away from the core team's campus and it served as the ambulatory primary care rotation location for pediatric residents at Atrium Health's Carolinas Medical Center (CMC) where LCSC-RC was housed. All CMC pediatric residents work at the PCC on a weekly basis. The team recruited the PCC advanced practice provider (APP) to serve as a local champion.

Accordingly, the measure of 10% increase in referrals for pediatric patients needing rheumatologist evaluation from four Atrium Health safety-net clinics to LCSC-RC by September 2021 had been modified to a 10% (17 patients) increase in accurate referrals from the PCC by December 2021. This number was determined by dividing 858 patients (baseline) by 5 years, equaling 171 patients in 1 year; therefore, 17 patients is a 10% increase.

Likewise, and due to the loss of one pediatric rheumatologist, the metric of 80% of all referred LCSC-RC patients seen within 30 business days from referral had been modified to 80% of all priority patients seen within 30 business days.

Priority population is defined as patients requiring ongoing rheumatology care, that is, pediatric patients <18 years of age referred from another provider and requiring the expertise of a pediatric rheumatologist for care of a perceived rheumatologic/autoimmune condition with symptoms to include any of the following criteria: ANA titer >1:320, specific joint swelling or pain, persistent fever, or rash. Priority patients were further subclassified into priorities 1, 2, and 3 (see Figure 1).

Our main tools were a referral tool, several visual aids including key information, and timely progress reports with the teams. LCSC-RC created a templated information referral tool in the EMR for primary care providers to complete, designed a triage tool for the receiving rheumatology team to use, and redesigned visit categories to reserve high acuity appointments in a previous project focused on increasing overall access to rheumatology care (6). The referral algorithm offers decision support to help referring providers understand referral requirements and provide specific information. The receiving referral coordinators then use the information to appropriately triage and schedule patients according to acuity. Incomplete forms trigger contact with the referring office and often include education. This allows for additional clarification of the requirements and ensures that patients are seen in a timely manner.

### 2.4 Change ideas

Change ideas included a focus on gaining primary care buy-in, team-to-team contact, training, and QI coaching. The team conducted multiple in-person visits at the PCC to develop a shared understanding of one another's work, to educate, and to test our tools. In October 2020, the LCSC-RC physician lead and the PCC APP met with PCC providers to introduce the project. The PCC providers requested decision support tools to facilitate appropriate identification of referral candidates. Returning the following month, the physician lead and APP introduced the PCC providers to the referral tool with a case study. PCC feedback endorsed the use of the referral tool combined with the case study and identified problems with removing outdated ICD 10 chronic diagnosis codes, their impact on workflow, and differences between attending physician use and resident physician use.

The resident developed visual aids to be displayed in three areas—on the wall, on computer keyboards, and on computer monitors—and in November 2020, the resident and APP determined that provider workrooms would be the most impactful locations. Flyers posted on the walls included triage priorities and instructions for following the referral pathway. Keyboard and monitor signs were small flags intended to reinforce behavior and provide EMR navigation support. The tools were placed in close proximity to one another. In December 2020, the physician lead shadowed the PCC providers to better understand their workflow and ensured that the visual aids were visible and in appropriate locations.

**Figure 2:**
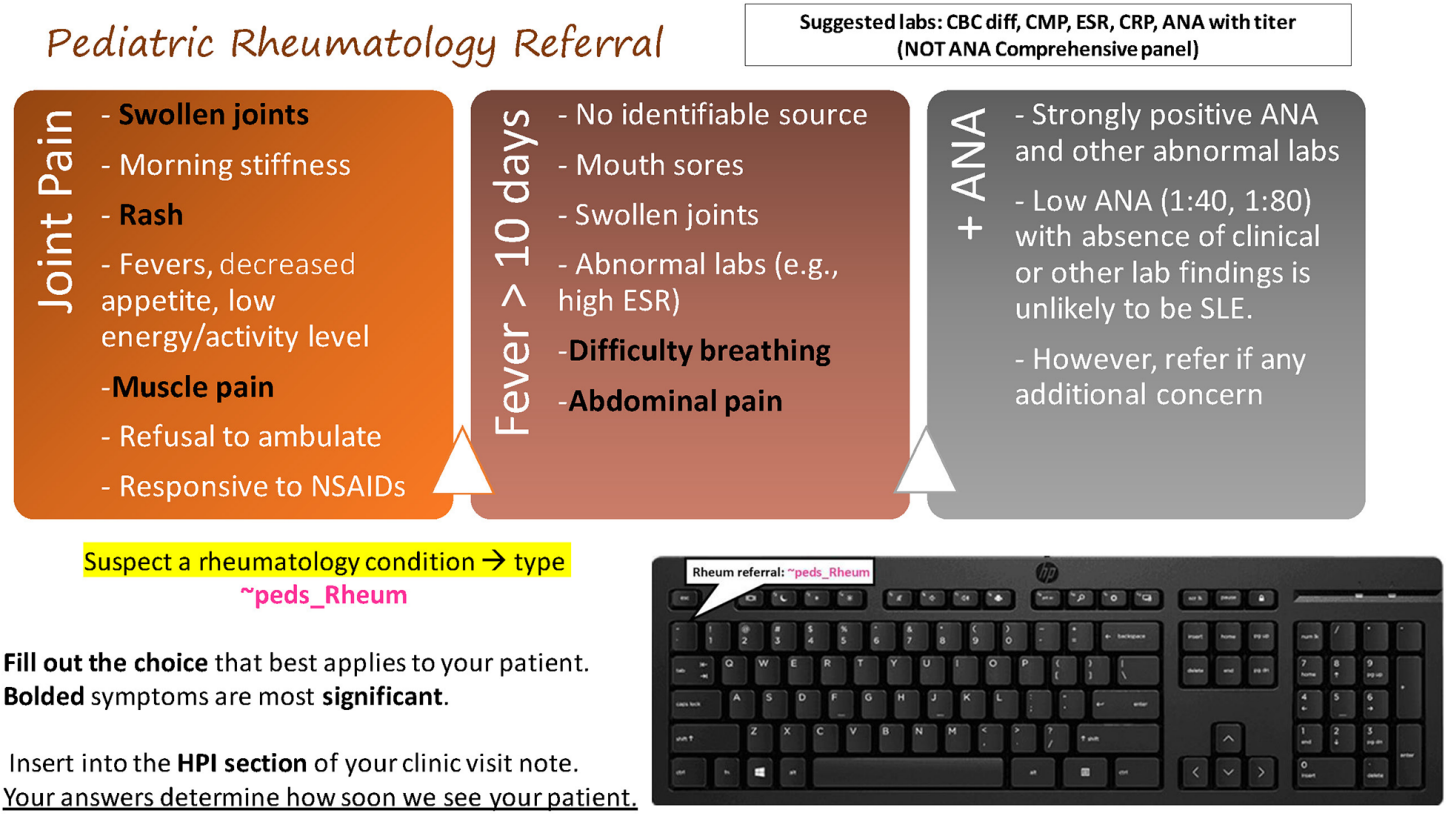
Visual Aids—Decision support for referring teams on the identification of symptoms likely connected with a rheumatologic condition. The boxes and instructions were in the form of printed flyers posted on the wall. The keyboard sign illustrates a reminder taped to clinic keyboards and computer monitors to reinforce behavior and provide EMR navigation support. Both tools were used simultaneously and placed in close proximity to each other.

In January 2021, the physician lead attempted to reconcile the ICD 10 chronic diagnosis codes, while the resident reintroduced the provider referral tool to her peers in February 2021. Based on feedback from the PCC providers, the project's resident and physician leads discovered that all providers needed more training to understand rheumatologic conditions and more durable, attention-grabbing visual aids. In March 2021, the visual aid posted on the workstation wall delineating referral-trigger symptoms and providing instructions for tool use was revised to incorporate bold fonts to highlight important items and different colors of background shading to separate symptom categories and was moved from behind computer monitors to a more visible space (Figure 2). The reminder taped to provider keyboards added pink font and was reinforced with stronger tape after it was discovered that the increasingly frequent sanitizing required during COVID made the original signs fall off quickly. Additional reminders were attached to the monitors.

The following months were dedicated to refreshing resident knowledge via their social media platform, and a division-wide resident-led grand rounds focused on the provider referral tool in April 2021. In the same month, the team turned their attention to previsit planning to better serve patients with language barriers. The triage tool of the rheumatology team was adjusted to capture language preferences, allowing an interpreter to be available for the appointment. Changes to the referral process for transportation needs and language barriers were embedded in a new EMR platform implemented in April 2022.

One innovation that required numerous PDSA cycles was the development of a culturally relevant patient story video paired with a referral tool tutorial video. The LCSC-RC physician lead had a teenaged patient from a Hispanic family who experienced a delayed diagnosis including multiple trips to the emergency room before being referred to LCSC-RC, resulting in a severe skin graft and hospitalization. The patient's story was particularly impactful, resulting in her serving as an advisor to the QI team. The team recorded this patient telling her story multiple times with intermittent testing at the PCC and with residents, improving scripting, focus, and audio/visual quality each time. The final product was combined with a previously produced instructional video of the physician lead explaining step by step how to access the referral tool through the EMR. This was shared electronically with the pediatrics leadership, office managers, and referral managers for the clinics involved and, through support from Atrium Health's marketing and communications office, with general pediatricians. These PDSA cycles spanned September 2021 through December 2021, with a special PDSA in September in which the resident shared the video with her resident peers and then conducted a survey on provider referral tool use to gauge knowledge retention, offering a gift card to incentivize survey completion. The provider lead also shared the video at a PCC monthly provider meeting in September.

## 3 Results

**Figure 3:**
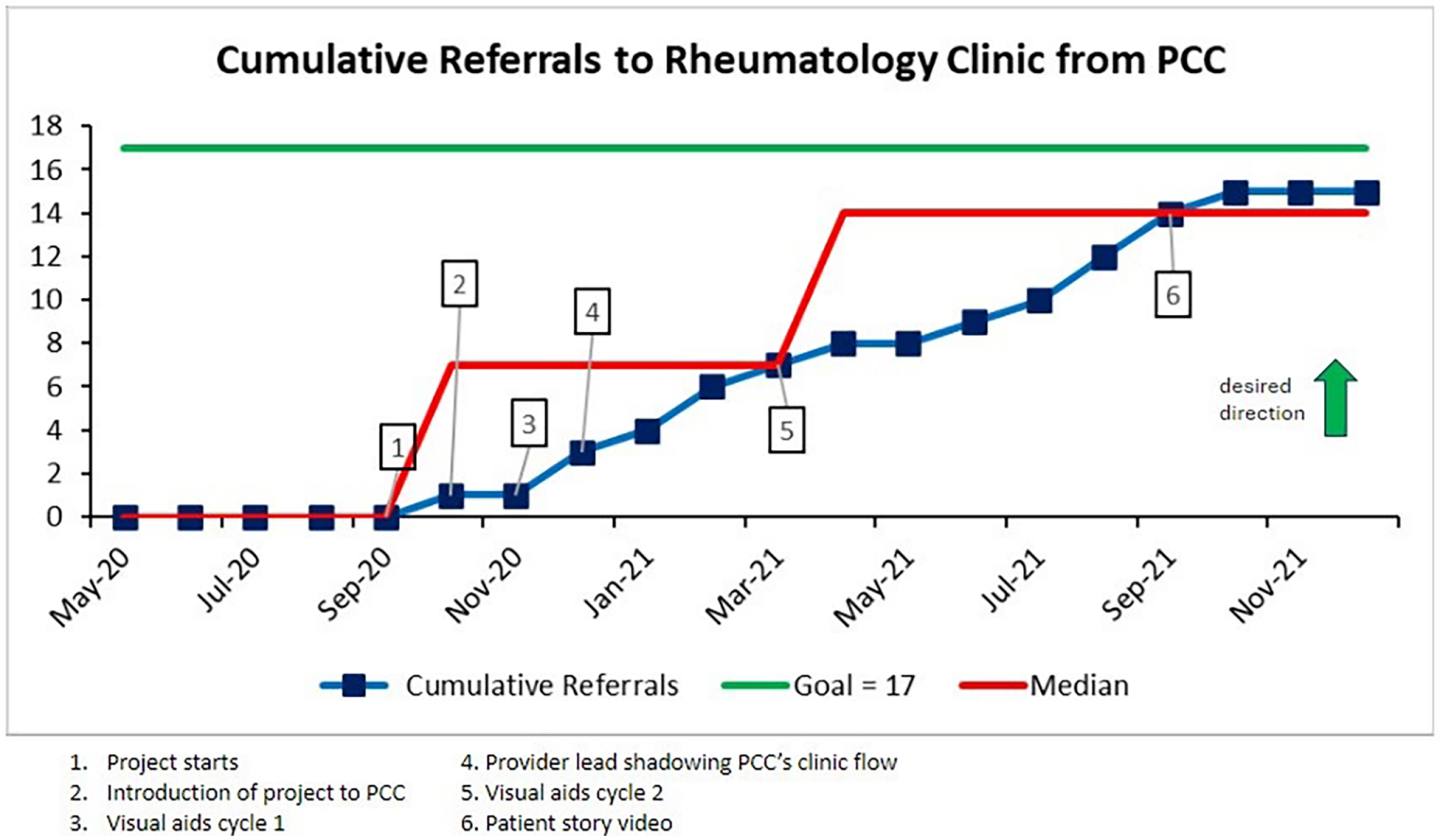
A run chart demonstrating cumulative referrals received from the target population. The referrals were evaluated on a monthly basis. The main interventions were noted. Two median shifts were observed based on the statistical rule of non-random variation of six data points above or below the median, ultimately reaching 15 total referrals.

As mentioned previously, the team had to modify the intended target population and narrow it down to only the PCC. The first measure of 10% increase in referrals needing rheumatologist evaluation for pediatric patients fell short by two patients, receiving 15 of 17 targeted referrals. Eight out of the 15 referrals completed the referral tool. We still considered this very significant since the PCC had not referred any patients from the targeted population in the 5 years prior to the project (Figure 3). Significant interventions that helped to achieve non-random variation included the provider lead visiting the PCC to shadow clinic workflow and address specific referring provider questions (December 2020), updating the visual tool (March 2021), and sharing the patient story video to residents and referring providers (September 2021).

**Figure 4:**
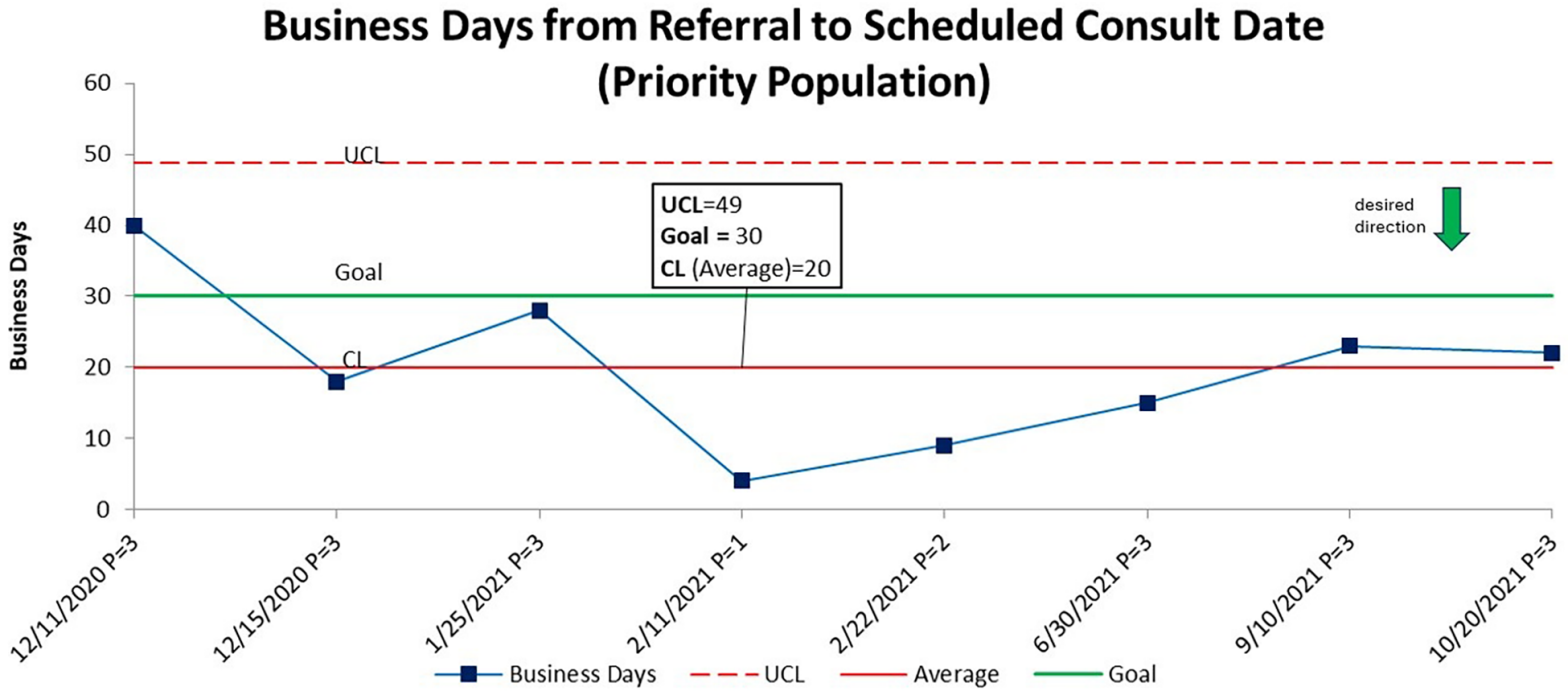
A statistical process control chart (SPC)—X chart demonstrating business days from referral to scheduled consult for priority populations. The center line (average) is stable at 20 business days. Each data point represents a patient and their level of priority. Only one patient missed the goal to be seen within 30 business days from referral to consult.

The second measure of 80% of all priority patients scheduled to be seen within 30 business days was achieved with an average of 20 business days from referral to consult (Figure 4). No special cause was observed. Other than the first patient referred, who was seen at 40 days from referral to consult, the goal of seeing referred patients within 30 days was surpassed and sustained at or above target for the remainder of the project.

Of the eight patients deemed priority, one was classified as priority 1, one as priority 2, and six as priority 3. Six were of Hispanic race and ethnicity, one was an African American, and one was an Asian, with the latter two having a non-Hispanic ethnicity. All eight patients had North Carolina Medicaid insurance. Their ages ranged from 7 to 16 years old, with four being males and four females. The final diagnoses given after a pediatric rheumatologist consult were hypermobility syndrome/hypermobile joints (5), pes planus (1), and systemic sclerosis (1). The 8^th^ remaining patient received a scheduled appointment for a referral complaint of right-hand pain and ANA titer of 1:640 but then canceled their appointment, thus a diagnosis could not be made. The process itself was successful.

## 4 Discussion

This project found new ways to apply existing data analytics by layering symptoms data onto zip code data to uncover significant gaps in care. The team applied communication strategies to improve provider education and patient care, such as combining a culturally relevant patient story with a tutorial on referral tool use; using a resident and advanced practice provider to influence their peers; and helping all members of the care team collaborate effectively to maximize limited personnel and resources. Due to improved communication, provider knowledge, previsit planning, and process efficiency, the number of patients seen from disadvantaged communities increased despite COVID-19 limitations and severe staff shortages.

The visual aids acted as critical tools. Safety-net clinics face continuity disruptions, experience very high volumes, and often have rotating resident learners providing care, making diagnosis or identification of specific symptoms more challenging. Team visibility and direct contact helped partners recognize symptoms and take appropriate action. Most primary care providers had limited exposure to pediatric rheumatology during training (9), and the Atrium community is often aware of LCSC-RC only if they have a patient with a previous rheumatologic disease or interact with the team.

Including the referral coordinator team was extremely important, as this group ensured the closure of the referral loop. They had a vested interest in making the process flow smoothly, because problems disrupted their own work. The PCC referral coordinators were highly responsive, ensured that the PCC providers completed the referral tool, and met with the LCSC-RC lead physician twice virtually and once in person.

The resident lead and PCC APP were instrumental in project success, serving as the eyes and ears of project implementation at the PCC. This would be the case any time, due to their consistent frontline work in the environment undergoing improvement, but they became irreplaceable when COVID restrictions were in place. The high volume of patients in a safety-net clinic can also make it difficult to test interventions and change processes because of the time constraints of the staff. We needed to be very strategic when building our relationships with key stakeholders in the practice and connecting with individuals who could help influence culture change and build goodwill, remove barriers, and facilitate tests of change and the adoption of successful strategies.

Resident feedback indicated that using a patient's story highlighting the challenges of a delayed diagnosis provided a meaningful connect to purpose, reminding everyone of the importance of this project.

Language and transportation access developed into an important component of the care package thanks to this work. Previously, in-person interpreters were often unavailable if they had not been prebooked, and online options often resulted in clinical disruption because of poor or dropped internet connections and subpar information delivery. The previsit planning involved in this project ensured that in-person interpreters were available during appointments, and the heightened demand for this service justified additional staffing for the language services division. Likewise, during the course of this work, the team recognized that some of the patient families encountered clinic attendance barriers due to transportation. As a result, grant funding from the rheumatology QI community via PR-COIN (Pediatric Rheumatology—Care & Outcome Improvement Network) now supports these needs through solutions such as gas cards and ride share vouchers. Transportation and language barrier questions are now a standard part of the referral triage process, with appropriate solutions provided as needed.

The COVID-19 pandemic presented a variety of challenges. With frontline staff already overwhelmed by pandemic patient needs, staff shortages impacted both clinics because of COVID infection and redeployment. Restricted clinic access made it more difficult to learn about the PCC's workflow and establish the best way to seamlessly incorporate the referral tool into their processes.

COVID highlighted the care discrepancies that marginalized populations endured. For example, many Atrium Health clinics adopted social distancing and telemedicine protocols to protect both staff and patients, such as completing paperwork online, collecting patient history via video, and having patients wait in their personal vehicles. Many of these strategies were problematic for PCC families who had limited technology access, who had language barriers, or who depended on public transportation. These restrictions can also impede the ability of a provider to understand the complicated nuances of rheumatologic symptoms.

Our attempt to reconcile ICD 10 chronic patient diagnosis codes in the EMR was a failed PDSA. While the best practice in the PCC is to regularly update the ICD 10 chronic patient diagnosis codes, we found that many PCC providers were not doing so consistently. The code list helps clarify potential diagnosis by illustrating patterns indicating chronic conditions. However, it proved extremely difficult for us to get the majority of providers to adopt the practice of updating the codes. Addressing this issue was beyond the scope of this project.

The importance of the primary care provider's understanding of symptom significance and referral process cannot be overstated for patients with social disparities, especially those with Medicaid insurance (a state-issued public insurance, typically provided to those who do not have or cannot afford private insurance). If a patient receives insurance from North Carolina Medicaid, they must receive a referral from a primary care provider before being able to see a specialist.

### 4.1 Limitations

A limitation in our project was the lack of completeness in approximately half of the referring providers’ submission of referral questions. As a result of limited information in seven referrals, the rheumatology team triaged patients with pain in multiple joints at a higher sensitivity rate. Ultimately, this led to a diagnosis of hypermobility syndrome/hypermobile joints, which was our most commonly identified diagnosis; while this syndrome is not a strictly rheumatologic condition, these patients benefited from a rheumatologic evaluation and may require ongoing subspecialist management (6). Another limitation of this project was that we identified only those patients whose homes were located in two public health priority zip codes and were referred by a safety-net clinic. The Mecklenburg County Public Health Department identifies four additional public health priority zip codes; patients residing in these areas could be included in future work (6). In addition, our data extraction exercise did not capture individuals who may suffer from social disparities but who do not live in zip codes specifically identified as “marginalized.”

### 4.2 Future directions

Our future plans include spreading this work to the other three safety-net clinics that were originally identified. All pediatric rheumatology clinics in our system are currently engaged in improving the accuracy of information relating to patient race and ethnicity in the EMR and ensuring its completeness, which is necessary to provide culturally appropriate resources and equitable access to tailored care. We received grant funding to conduct a primary qualitative research study using interviews and surveys with families from safety-net clinics to explore their experiences and barriers to care. Patients will provide insight into improving processes, contribute ideas for a patient-centered referral process, and participate in developing health literate printed materials and/or scripting. In the future, we plan to create additional videos of patients’ journeys. We would like to work with patients to develop a health “passport” to facilitate navigation of their own health, the healthcare system, and complex conditions. This passport will be especially helpful for patients who see providers from multiple healthcare systems with different electronic medical record platforms. This passport will become a printed document that they will carry when returning multiple times to a provider for a variety of symptoms or entering a new area within the system.

Sustainability of this work was rendered challenging due to the collaborating safety-net PCC undergoing leadership change, nursing staff turnover, EMR transition, change in the referral process, and referral coordinator roles being redefined. The project required a re-evaluation to find a more sustainable path forward, and is currently undergoing a refresh. Specific activities include new resident involvement and pediatric leadership support to design innovative tools. Ongoing work in this renewed environment includes patient interviews, new visual tools, new EMR tools (smart phrase and ambulatory referral order), and regrouping with other community-wide health systems that have also acquired new EMR systems.

This project and its predecessor (6) illustrate that by leveraging clinical information systems to enhance gap identification to improve access to rheumatologic care, teams can continually drive and refine next steps and create a constant feedback loop to increase focus and knowledge in safety-net clinics for vulnerable populations. The lack of an efficient process and delayed diagnosis exacerbate disparities and impact outcomes, quality of life, patient/staff satisfaction, affordability, and resource allocation. With pediatric rheumatologists in such short supply, this work is crucial to ensuring that those children most in need receive timely access to subspecialist care. Navigating the future of this discipline requires flexibility to adapt and a willingness to embrace change, both of which benefit from and contribute to a learning health system. Sharing the strategies and lessons learned from this project can improve outcomes across the pediatric primary/subspecialty care spectrum.

## Data Availability

The original contributions presented in the study are included in the article/Supplementary Material, and further inquiries can be directed to the corresponding author.
